# Neocytolysis: How to Get Rid of the Extra Erythrocytes Formed by Stress Erythropoiesis Upon Descent From High Altitude

**DOI:** 10.3389/fphys.2018.00345

**Published:** 2018-04-05

**Authors:** Heimo Mairbäurl

**Affiliations:** Medical Clinic VII, Sports Medicine, Translational Lung Research Center, German Center for Lung Research, University Hospital Heidelberg, Heidelberg, Germany

**Keywords:** high altitude medicine, erythropoiesis, reticulocytes, hemoglobins, total hemoglobin mass, antioxidant capacity, hypoxia

## Abstract

Neocytolysis is the selective destruction of those erythrocytes that had been formed during stress-erythropoiesis in hypoxia in order to increase the oxygen transport capacity of blood. Neocytolysis likely aims at decreasing this excess amount of erythrocytes and hemoglobin (Hb) when it is not required anymore and to decrease blood viscosity. Neocytolysis seems to occur upon descent from high altitude. Similar processes seem to occur in microgravity, and are also discussed to mediate the replacement of erythrocytes containing fetal hemoglobin (HbF) with those having adult hemoglobin (HbA) after birth. This review will focus on hypoxia at high altitude. Hemoglobin concentration and total hemoglobin in blood increase by 20–50% depending on the altitude (i.e., the degree of hypoxia) and the duration of the sojourn. Upon return to normoxia hemoglobin concentration, hematocrit, and reticulocyte counts decrease faster than expected from inhibition of stress-erythropoiesis and normal erythrocyte destruction rates. In parallel, an increase in haptoglobin, bilirubin, and ferritin is observed, which serve as indirect markers of hemolysis and hemoglobin-breakdown. At the same time markers of progressing erythrocyte senescence appear even on reticulocytes. Unexpectedly, reticulocytes from hypoxic mice show decreased levels of the hypoxia-inducible factor HIF-1α and decreased activity of the BCL2/adenovirus E1B 19 kDa protein-interacting protein 3 (BNIP3), which results in elevated mitochondrial activity in these cells. Furthermore, hypoxia increases the expression of miR-21, which inhibits the expression of catalase and thus decreases one of the most important mechanisms protecting against oxygen free radicals in erythrocytes. This unleashes a series of events which likely explain neocytolysis, because upon re-oxygenation systemic and mitochondrial oxygen radical formation increases and causes the selective destruction of those erythrocytes having impaired anti-oxidant capacity.

## Introduction

Adjusting amount and function of erythrocytes is required in a variety of situations, the most obvious being the decrease in Hb after birth and the slow replacement of erythrocytes containing HbF with ones with HbA after birth (Terrenato et al., 1981). Altered blood distribution among compartments seems to induce a reduction in the total mass of erythrocytes in microgravity (Alfrey et al., 1996). Many diseases are associated with anemia by removing damaged erythrocytes (e.g., Rifkind, 1966). Although destruction likely occurs by different mechanisms, all might be classified into the non-specific term “erythrolysis.” Yet another mechanism might explain the destruction of erythrocytes when highly polycythemic high altitude natives descent from high altitude (Merino, 1950). This review addresses targeted destruction of erythrocytes upon return to sea level, which lowlanders form during staying at high altitude for a limited time. Because in particular these newly formed cells seem to be removed, their destruction is called “neocytolysis” (Alfrey et al., 1997). For better understanding selectivity of destruction, biochemical characteristics, and mechanisms of cell destruction of erythrocytes formed under normal and stress conditions will be summarized.

## Adjustments of oxygen transport to hypoxia at high altitude

Oxygen supply to tissues is impaired at high altitude because decreased oxygen content of inspired air resulting in a mild decrease in arterial oxygen saturation (SaO_2_) at moderate altitudes (~92% at 2,500 m) but much more pronounced at higher altitudes (~83% at 4,500 m) because of the sigmoidal shape of the oxygen dissociation curve (Mairbäurl and Weber, 2012).

Arterial oxygen content (CaO_2_) varies during a sojourn at high altitude (Rasmussen et al., 2013): CaO_2_ is decreased upon acute ascent to high altitude, increases slightly within a few hours because of ventilatory acclimatization and respiratory alkalosis, increases further within days because of decreased plasma volume and increased hematocrit, and increases even further within weeks to months at high altitude because stimulated erythropoiesis increases total hemoglobin (tHb) reaching even pre-altitude values (Calbet et al., 2003).

Importantly, only CaO_2_ gets normalized but not PaO_2_, which is the main driving-force for oxygen diffusion to tissues, which therefore remain to some extent oxygen-limited. This seems to be well tolerated because at some point during a long-term stay at high altitude tHb reaches a stably-elevated value (Hurtado et al., 1945; Merino, 1950; Reynafarje et al., 1959). Performance is not improved equally because of impaired O_2_-diffusion (Calbet et al., 2003; Bärtsch and Swenson, 2014).

Stimulation of erythropoiesis at high altitude depends on HIF-2α and erythropoietin (EPO) (Haase, 2013) and adjustments of iron metabolism (Hentze et al., 2010). EPO increases rapidly upon exposure to hypoxia; the magnitude depends on the degree of hypoxia (Eckardt et al., 1989) following a semi-logarithmic function (Wenger and Kurtz, 2011). After this initial increase EPO decreases significantly to reach a steady state level that remains significantly above normoxic values, while one is still exposed to hypoxia (Wenger and Kurtz, 2011). Yet, the rate of formation of new erythrocytes stays elevated provided there is sufficient supply with iron. Iron uptake seems to peak after approximately 4 days at high altitude, reticulocytes have their highest values after approximately 7 days in hypoxia (Siri et al., 1966). This pattern of changes has been named the “EPO-paradox” (Milledge and Bärtsch, 2014). It is likely caused by a shift from hematopoietic stem cells toward erythroid progenitor cells associated with up-regulation of the erythroid transcription factor GATA-1, which stimulates EPO-receptor expression (Li et al., 2011), resulting in an increased EPO-sensitivity and sustained erythropoiesis even at lower EPO. EPO receptors on progenitor cells are gradually lost during differentiation, and EPO-receptor density is very low in circulating mouse erythrocytes (Mihov et al., 2009).

The rapid increase in reticulocyte count, which is commonly observed, is likely caused by increased bone marrow blood flow and immature release of red cells (Aoki and Tavassoli, 1981). These immature cells differ in properties from reticulocytes produced under stress-free conditions, similar to what has been found in thalassemia (Rivella, 2009).

At moderate altitudes 2 weeks do not result in an increase in Hb in athletes (Friedmann et al., 1999), and stays longer than 3 weeks seem to increase total red cell volume by 60–250 ml/week (Sawka et al., 2000). Natives to high altitudes in the Andes have an increased total Hb, and their blood volume in increased by approximately 20% (Hurtado, 1964; Sánchez et al., 1970). Similar values are observed in sojourners after weeks to months at high altitude.

## Maturation of erythroid precursors

The viability of committed erythroid progenitor cells downstream of the erythroid differentiation depends on the presence of EPO by supporting survival of erythroid precursors, which stimulates proliferation (Kimura et al., 2000). This process depends on cKit and EPO-receptors on the progenitor cells (Fisher et al., 1994; Wenger and Kurtz, 2011) and other growth factors all of which cause tyrosine-phosphorylation by Janus-kinase JAK2 (Klingmüller, 1997). JAK2 phosphorylates the EPO-receptor and the signal transducer and activator of transcription 5 (STAT5), which blocks apoptosis by inhibiting the Forkhead-Box-Protein O3 (FOXO3)-dependent pro-apoptotic pathways (Wojchowski et al., 2006). There is also an inhibition of the death receptor Fas and its ligand FasL in splenic erythroid cells, which promotes survival (Koulnis et al., 2011). The BNIP3-ligand (BNIP3L; Nix) controls mitochondrial autophagy (Sandoval et al., 2008). Thus, by the action of EPO, dividing progenitor cells are not destroyed resulting in increased production of normoblasts and release of reticulocytes into circulation (Wenger and Kurtz, 2011). In addition, phosphorylation of STAT3 increases the expression of antioxidant enzymes such as superoxide dismutase (SOD) and glutamin-cystein antiporter xCT while at the same time decreases the expression of proteins of the mitochondrial electron transfer chain (Linher-Melville and Singh, 2017). Together these two processes protect from hypoxia-induced mitochondrial oxygen radicals (ROS) (Chandel and Schumacker, 2000), which might impair erythropoiesis. Hypoxia also causes a HIF-1α and BNIP3-dependent decrease in mitochondrial activity, which further reduces ROS formation (Semenza, 2008). Therefore, during acute hypoxia, when mitochondrial adjustments have not yet been established, hypoxia likely increases mitochondrial ROS production, whereas hypoxia-adapted cells seem to produce less ROS (Chandel et al., 1997). However, there is also evidence that hypoxia might down-regulate the expression of anti-oxidant enzymes such as catalase (Song et al., 2015). If this were the case, then one might speculate on a decreased anti-oxidant activity in hypoxia-adjusted cells and increased vulnerability to oxidant damage. This issue requires clarification.

## Stress-erythropoiesis

While normal steady-state erythropoiesis produces cells at a nearly constant rate, situations of acute tissue hypoxia such as blood loss, hemolysis, and elevated erythropoietin dramatically increase the rate of erythrocyte production and the rapid appearance of newly formed cells in circulation (“stress-erythropoiesis”). Most experimental evidence comes from work on mice, whereas evidence for human equivalents is sparse. It has to be noted that the mouse model most often used is phenylhydrazine-induced lipid-peroxydation and hemolytic anemia (Jain and Subrahmanyam, 1978), a quite “un-physiological” system.

In the acutely anemic mouse some of the EPO-induced progenitors migrate to the spleen (there may also be resident, self-renewing ones), with much enhanced maturation (Paulson et al., 2011). This is based on the finding that erythroid burst forming units (BFU-E) from the spleen differ from bone marrow BFU-E in that they form larger colonies and that the only growth factor required is EPO, whereas in the bone marrow additional burst-promoting factors are required (Valtieri et al., 1989). Bone morphogenic protein-4 (BMP4), who's expression depends on HIF-2α (Wu and Paulson, 2010), induces the expansion of BFU-E in the spleen to produce specialized resident stress erythroid progenitors (Paulson et al., 2011). It is interesting to note that hypoxia strongly stimulates the expansion of spleen-derived BFU-E (Perry et al., 2007). There is only indirect evidence for similarities between murine and human stress erythropoiesis. In acute anemia human stress erythropoiesis exhibits similarities to fetal erythropoiesis (Paulson et al., 2011), because a higher proportion of blood progenitor cells than typical bone marrow derived cells contains HbF. Hypoxia of cultured progenitor cells from patients with sickle cell disease and thalassemia also induces the production of HbF cells. A moderate increase in HbF-containing erythrocytes has also been found in humans after a 17-day stay at altitudes above 3,100 m (Risso et al., 2012). Thus it was speculated that this type of stress progenitors resemble the spleen-derived stress BFU-Es of the mouse model.

The marrow transit time of ^59^Fe was shortened in mice made anemic by phlebotomy, and there was an inverse relation between transit time and the degree of anemia (Hillman, 1969). Cells appear to be released immaturely indicated by larger size, increased reticulum content, iron uptake, membrane ion transporter activity, and increased density of transferrin receptors (TfR; CD71) on the plasma membrane (summary in Rhodes et al., 2016). TfR could be detected on circulating erythrocytes longer than during normal erythropoiesis. This indicates that the rate of maturation is similar in stress and normal erythropoiesis, but that maturation of circulating stress reticulocytes takes longer because of their immature release (Al-Huniti et al., 2005; Rhodes et al., 2016).

## Neocytolysis

Neocytolysis is the selective destruction of the youngest population of erythrocytes in blood, just after they had left the bone marrow, and it is thought to bring an elevated erythrocyte mass (e.g., after a stay at high altitude) back to normal (Alfrey et al., 1997). It seems to occur in a variety of different situations (Alfrey et al., 1997; Harris and Epstein, 2001; Song et al., 2017). Neocytolysis appears to be caused not simply by discontinuing stress erythropoiesis but by “controlled” processes (for a recent review see Risso et al., 2014). However, indications for neocytolysis after return from high altitude hypoxia are weak.

Merino noted disappearance of polycythemia within a few days after return from high altitude and explained it by reduced erythropoiesis (Merino, 1950), which has been indicated experimentally by decreased iron incorporation in high altitude natives on traveling to low altitude (Huff et al., 1951). Also the reticulocyte number decreased. Greater “blood destruction” was indicated by increased plasma bilirubin and urobilinogen excretion (Merino, 1950).

Pace et al. based the discussion of neocytolysis (Pace et al., 1956) on a normal erythrocyte disappearance rate of 0.0083 per day (experimentally determined from the rate of removal of transfused erythrocytes; Callender et al., 1945). Erythrocyte count and Hb decreased at a rate of 0.011 per day upon return to sea level after an expedition to the Himalayas (Pace et al., 1956). This was interpreted to be caused by decreased rate of erythropoiesis and increased “erythrolysis,” but also by restoring the decrease in plasma volume that occurs at high altitude (Siebenmann et al., 2017).

Rice et al. studied polycythemic residents of Cerre de Pasco (4,380 m) upon travel to sea level and found a decrease by 9% in erythrocyte mass within 3–7 days after descent, a rapid decrease in EPO, and increased bilirubin. Changes did not occur in three subjects receiving EPO upon descent (Rice et al., 2001).

Polycythemia by itself shortens erythrocyte survival (Bogdanova et al., 2007). Risso et al. (Risso et al., 2007) separated age-fractions of erythrocytes by density gradient centrifugation from blood of mountaineers 1 day after return from a mountaineering expedition and found that the youngest fraction had disappeared. Cells had acquired a “senescent phenotype” indicated by decreased levels of expression of the integrin associated protein (CD47), of the complement decay accelerating factor (DAF; CD55), and of protectin (membrane inhibitor of reactive lysis; CD59), which may be an indication of increased susceptibility to phagocytosis (Risso et al., 2007). However, only one time-point had been studied, and results may be flawed by the fact that the descent itself had lasted almost 1 week.

### Mechanisms causing neocytolysis

Results on three subjects (Rice et al., 2001) suggest that the withdrawal of EPO might be responsible for the destruction of erythrocytes produced in hypoxia. It is thought that increased EPO favors the expression of CD55 and CD59 such as observed in EPO-treated patients with renal anemia (Ohi et al., 2003), which protects from destruction by macrophages, whereas EPO withdrawal decreases expression and coincides with reappearance of anemia (Ohi et al., 2003), resulting in a picture comparable to that observed with EPO-withdrawal and treatment upon descent (Trial and Rice, 2004; Risso et al., 2014). This seems to be in line with the dependency of erythrocyte destruction on EPO and EPO-receptors in polycythemia of a gain-of-function mutation of the EPO-receptor (Divoky et al., 2016).

Another line of evidence suggests a role for altered antioxidant capacity. Erythrocytes exposed to anoxia have a decreased antioxidant capacity, indicated by decreased glutathione, nicotine-adenine dinucleotide (phosphate) (GSH, NADPH, and NADH, resp.) redox couples and reduced membrane thiols (Rogers et al., 2009). This has been explained with a re-direction of glycolytic flux toward the synthesis of 2,3-diphosphoglycerate (2,3-DPG) and binding to deoxygenated Hb, as well as altered glycolytic activity caused by competition of binding between deoxygenated Hb and glycolytic enzymes to band 3 (Weber et al., 2004), which also results in decreased NADPH and GSH formation (Rogers et al., 2009). Though this mechanism might adversely affect erythrocyte survival during high altitude hypoxia, it should rapidly be reversed upon descent thus protecting mature erythrocytes from oxidative stress. Erythropoiesis in hypoxic mice results in erythrocytes with a decreased anti-oxidative capacity because of decreased expression of catalase due to elevated levels of the micro-RNA miR-21 (Song et al., 2015). Anti-oxidative enzymes are typically high in young erythrocytes and decrease rapidly with aging (Bartosz and Bartkowiak, 1981). In the mouse model hypoxia during maturation increases mitochondrial activity in erythroid precursors due to suppression of HIF-1α and decreased BNIP3L. It was argued that mitochondrial ROS production would increase in normoxia and cause neocyte destruction (Song et al., 2015). This is in sharp contradiction to results showing HIF-1α-induced increase in mitophagy and reduction of mitochondrial mass in a variety of cell types thought to protect from increased ROS formation and cell destruction (Zhang et al., 2008) because hypoxic mitochondria produce more ROS than normoxic ones (Chandel et al., 1998; Chandel and Schumacker, 2000; Levraut et al., 2003). This issue awaits clarification.

Interestingly, it was found that treating mice with polyethylene glycol-conjugated catalase, which is not taken up by reticulocytes and mature erythrocytes, as well as treatment with the anti-oxidant N-acety-cysteine increased reticulocyte half-life and prevented the reduction in hematocrit (Song et al., 2015). This result contradicts the above described argument for a role for elevated mitochondrial but argues for systemic elevation of ROS which damage hypoxia-derived neocytes, a mechanism similar to re-oxygenation injury known from many organs, e.g., the lung (Pak et al., 2017).

## Destruction of senescent erythrocytes

The average life-span of mature erythrocytes is 100–130 days. The destruction rate amounts to approximately 1% per day. Senescent erythrocytes are removed by the reticulo-endothelial system, mainly in the spleen, where cells are tested for functionality and are sequestered “if they don't pass the test” (Rifkind, 1966). Random hemolysis of pre-senescent erythrocytes is negligible in humans but amounts to 0.5–1% per day in mice and rat (Landaw, 1988). Loss of erythrocytes is balanced by a production rate of ~160 × 10^6^ erythrocytes per minute in humans.

Interestingly, erythrocytes produced in hypoxic stress appear to have a shortened life span in some species, which is caused by increased random hemolysis and accelerated senescence (Fryers and Berlin, 1952). Every doubling of the erythrocyte production rate resulted in a 3.5% reduction in survival in rat (Landaw, 1988). Mechanisms seem to be intrinsic to erythrocytes, because cross-transfusion of newly formed cells into normoxic animals did not improve survival (Landaw, 1988). This is in line with reports on elevated levels of breakdown-products of heme in high altitude residents (Merino, 1950). It is further supported by an approximately 25% increase in the ^59^Fe disappearance rate from blood in Peruvian high altitude relative to sea-level residents (Huff et al., 1951) indicating increased production due to accelerated sequestration to maintain stably elevated Hb.

## Consequences of neocytolysis and accelerated senescence

If in fact erythrocytes produced by hypoxic marrow have characteristics limiting their survival upon return to normoxia, then hemolysis will affect different age-cohorts of erythrocytes depending on the duration of the sojourn and on the altitude (Rasmussen et al., 2013). After a sojourn at moderately high altitude for a few weeks as done by athletes for continuous or intermittent altitude training with the goal of increasing oxygen transport capacity and sea-level performance (Levine and Stray-Gundersen, 1997; Stray-Gundersen and Levine, 2008), only a small fraction of newly released erythrocytes will be found in circulation (Rasmussen et al., 2013; Garvican-Lewis et al., 2016). In fact, athletes had an elevated fraction of immature reticulocytes with high expression of TfR after returning from training at 1,905 m, which fell to sub-baseline values by day 9, and returned to baseline on day 16 after the training camp (Nadarajan et al., 2010). Similarly, increased ferritin levels were found post-altitude-training (Garvican et al., 2012). These changes are consistent with neocytolysis. However, there were no or only minor changes in tHb within 1 or 2 weeks after return from training at 2,300 m, during which total Hb had increased by ~8% (Prommer et al., 2010; Garvican et al., 2012; Wachsmuth et al., 2013a,b), which may indicate decrease in erythrocyte mass by decreasing erythropoietic activity and by random loss, but a minor contribution of neocytolysis. In contrast, polycythemia after a stay at 5,260 m was reverted already within 1 week after descent indicating neocytolysis (Ryan et al., 2014). This may indicate that neocytolysis plays a minor role after a stay at moderate altitude, and that athletes performing altitude training may take advantage of a slightly increased oxygen carrying capacity at low altitudes for several weeks.

The situation is different for long-term sojourners and high altitude residents returning to low altitudes, because all their erythrocytes in circulation may have a decreased anti-oxidant capacity. Thus, elevated ROS will not only lyze young erythrocytes, but also random destruction might be increased and shorten erythrocyte life span. Hemolysis will progress until all erythrocytes with high altitude characteristics will be replaced by normoxic ones having increased antioxidant capacity. This process might cause a severe hemolytic strain, which might be even more pronounced in polycythemic individuals with chronic mountain sickness.

## Conclusion and perspectives

There are indications of destruction of erythrocytes formed during exposure to hypoxia upon return to normoxia. Mechanisms are unclear and might include withdrawal of EPO and impaired defense against increased ROS. Experiments are needed to better define the mechanisms causing the fast and selective removal of neocytes formed in situations of stress erythropoiesis and to clearly distinguish between selective destruction and random loss.

Another aspect to be clarified concerns the use of the term “neocytolysis,” which implies the destruction selectively of erythrocytes formed in an acute and transient situation of stress erythropoiesis. Thus this term cannot be used to describe the destruction of a fraction of erythrocytes produced during long-term or even life-long exposure to such a stress environment such as fetal life and being an altitude native. In those situations it needs to be sorted out whether just erythrocytes newly released from the marrow are removed, or whether all circulating cells are more susceptible to random loss, and why.

## Author contributions

The author confirms being the sole contributor of this work and approved it for publication.

### Conflict of interest statement

The author declares that the research was conducted in the absence of any commercial or financial relationships that could be construed as a potential conflict of interest. The reviewer IB and handling Editor declared their shared affiliation.
